# “It’s a delicate balance”: clinicians’ experiences of providing heroin-assisted treatment

**DOI:** 10.1186/s12954-024-01135-2

**Published:** 2024-12-31

**Authors:** Rune Ellefsen, Silvana De Pirro, Vegard Haukland, Linda Elise Couëssurel Wüsthoff, Espen Ajo Arnevik

**Affiliations:** 1 Unit for Clinical Research on Addictions, Oslo University Hospital Health Trust, PB 4959 Nydalen, Oslo, 0424 Norway; 2 Department of Addiction Medicine, Haukeland University Hospital, Østre Murallmenningen 7, Bergen, 5012 Norway; 3 Norwegian Centre for Addiction Research, Faculty of Medicine, University of Oslo, Kirkevegen 166, Building 45, Oslo, 0407 Norway

**Keywords:** Diacetylmorphine, Injectable opioid agonist treatment, Clinical experience, Patient-centered care, Treatment provision, Qualitative research, Nursing

## Abstract

**Background:**

Little attention has been paid to the experiences of clinicians and health personnel who provide heroin-assisted treatment (HAT). This study provides the first empirical findings about the clinicians’ experiences of providing HAT in the Norwegian context.

**Methods:**

23 qualitative interviews were conducted with 31 clinicians shortly after HAT clinics opened in Norway’s two largest cities: Oslo and Bergen. By inductive thematic analysis of interview transcripts, we identified what research participants experienced and viewed as the chief rewards and challenges of providing HAT. The study aimed to offer an overview of these key rewards and challenges, with insights potentially transferable to HAT programs internationally.

**Results:**

Participants experienced three aspects of providing HAT as particularly rewarding, and three as most challenging. The rewarding aspects were observing harm reduction outcomes; providing holistic care; and having a positive clinic milieu and patient–clinician relationships. The challenging aspects were dosing and overdose risk; rule enforcement and aggression management; and the difficulty of initiating treatments beyond medication and harm reduction. The rewarding and challenging aspects of providing HAT overlapped and were at times contradictory, thus reflecting the duality and tensions in clinicians’ work to provide HAT. The challenges were reported to vary between patient subgroups, according to their degree of instability. The most unstable patients were seen as involving more difficulties as regards the challenging aspects of HAT. Participants expressed uncertainty about HAT’s utility for a small group of the most unstable patients.

**Conclusion:**

While studies about clinical experiences of HAT have usually examined individual or limited aspects of treatment provision, this study provided an overview of the main aspects of the rewards and challenges of providing HAT. Importantly, it also showed the tensions between these overlapping and sometimes contradictory aspects of HAT provision. Because a positive patient–clinician relationship is crucial to patient satisfaction and treatment outcomes in HAT, the provision of training for clinicians on navigating the inherent tensions of HAT provision, nurturing therapeutic alliances with patients, and managing their role as gatekeepers to medical heroin and valuable services, seem particularly important for ensuring that care is patient-centered and staff are adequately supported.

## Background

Heroin-assisted treatment (HAT) is administered to patients with opioid use disorder (OUD), and primarily patients who have not benefited sufficiently from traditional opioid agonist treatment (OAT). HAT is considered an evidence-based treatment option for this vulnerable group of patients for whom the risk of fatal overdoses is substantial when out of treatment [1, 2]. HAT involves supervised self-administration of medical-grade heroin (diacetylmorphine) in designated clinics, where psychosocial support is often available. Permanent HAT programs have been established in Switzerland, the Netherlands, Germany, Denmark and Canada, while temporary trials are ongoing in Scotland, Luxembourg and Norway [3]. This study was conducted by independent scholars as part of a government-initiated evaluation of the Norwegian five-year HAT trial project, which began in 2022 with clinics being opened in the country’s two largest cities: Oslo and Bergen. The aims of Norwegian HAT and evaluations of it, together with patient characteristics in this country are reported elsewhere [3–5].

HAT seeks to meet patients’ medical needs, and the provision of medical heroin with a short half-life generally results in highly intensive and regulated treatment programs. While HAT exists in several countries, such programs have different configurations. In Norway, HAT is part of OAT in specialized health care services and is institutionally placed in hospitals. This contrasts with Danish HAT, which is positioned in the municipality, alongside social-care services. The Norwegian HAT trial project is funded through the national health-care budget [6], while HAT in the United Kingdom (UK) has been funded by commissioning partners across the criminal justice and health-care sectors [7]. HAT in Canada differs from the European HAT context, as injectable hydromorphone is provided at some sites and the country is undergoing an opioid crisis [8]. The contextual specifics of Norwegian HAT are thus relevant to interpreting the results reported below and their transferability to other countries.

Randomized controlled trials and systematic reviews have found that HAT has desirable patient outcomes (e.g., reduced overdose risk, improved quality of life) and societal benefits (e.g., reduced burden on the criminal justice sector) [8–10]. Most HAT research has been quantitative, though some qualitative studies have investigated patients’ treatment experiences [11–15]. In contrast, limited attention has been paid to the roles and experiences of those who provide HAT [16]. This study seeks to help fill that gap.

Clinicians play a crucial role in both HAT and OAT, based on their abilities to form therapeutic alliances with patients, and in their attitudes toward patients, which may significantly impact patients’ experience of HAT and its outcome [17–19]. If clinicians believe that the treatments they provide are important for patients, they are more likely to have higher levels of commitment to these patients [16, 20]. Research on the involvement of clinicians and health-care professionals in OATs like HAT has been limited, and scholars have thus called for greater investigation of this topic [21]. Evidence of HAT provision from the clinicians’ perspective is generally scarce [7, 22].

Qualitative research to date has shown that in the experiences of Canadian HAT clinicians, inappropriate clinic facilities are the greatest obstacle to providing quality treatment [16]. Clinicians in Switzerland, the Netherlands, Germany, and the UK report concerns about overdose risks from intoxicated HAT patients receiving medical heroin [22]. Danish HAT staff expressed similar concerns [23]. Studies also show aspects of HAT that clinicians view positively. Spanish HAT nurses reported that offering physical care and guidance on injections created opportunities to discuss important patient issues, like the patient’s non-prescribed drug use [24]. UK clinicians observed gradual changes in patients’ outlook and appearance after they entered HAT, providing reassurance about the treatment’s utility [1]. Clinicians have also emphasized that it is important to them that HAT involves more than providing medical heroin [7].

Several studies have documented efforts by HAT clinicians to remain flexible about patients’ needs to reduce the intensity of this treatment regime [7]. In attempts to provide patient-centered care, clinicians have sought to ensure that patients have “power and a more equitable relationship” with staff [16]. Clinicians have thus used creative means to foster patient autonomy by “advocating for more individualized care” in a setting which is often highly restrictive [25].

In both HAT and traditional OAT, there is an inherent tension between providing patient-centered care, which seeks to tailor the treatment to patients’ needs, and program-centered care, which prioritizes program goals and protocol adherence [26]. Although OAT has traditionally been program-centered, patient-centered approaches are becoming widespread [27, 28]. These involve principles of collaboration with patients to develop flexible treatment goals, including orientation toward clinical and nonclinical outcomes and quality of life, rather than abstinence [27]. HAT involves elements of both patient- and program-centered care, a distinction that is useful for understanding clinicians’ experience regarding this treatment [see also 25].

The goal of this qualitative study was to investigate the clinicians’ experiences of providing HAT in Norway. By inductive thematic analysis of 23 transcribed interviews with 31 clinicians, we identified what clinicians saw as the most important rewards of, and challenges involved in, providing this treatment. While previous studies examine singular or delimited aspects of the clinical experiences of HAT provision, this study aimed at providing an overview of what clinicians see as being most positive and most challenging about HAT.

### Methods

#### Setting

Norwegian HAT provides supervised administration of medical heroin, with up to two doses per day by injection or tablets. Additionally, patients received methadone or 12-hour slow-release oral morphine (SROM) as bridge medication overnight. HAT clinics are sited in hospitals, but their patients do not have greater access to specialized health care services compared with the general public. Patients are referred to other specialist health services (i.e. for specific somatic or psychiatric needs) when relevant. The clinics also collaborate with community services.

During data collection, the number of patients was about 10–20 in Bergen and 20–40 in Oslo, and it had increased to 109 (34 in Bergen and 66 in Oslo) in November 2024, which is still significantly lower than the original estimate of up to 300 patients [3]. Additionally, the number of staff has slightly increased since the data collection period.

#### Participants and data collection

In-depth qualitative interviewing was seen as the best suited method to capture clinicians’ subjective experiences and views. Research participants were recruited either by contacting clinicians individually or through open invitations to HAT staff to join a group interview. Between 80 and 90% of the Oslo and Bergen staff participated, following administration of informed consent. To protect participants’ privacy, their specific roles, professions and the clinics in which their interviews took place are not specified in the results below. All participant names used are pseudonyms.

Data were collected within the first 14 months of HAT operation in Norway. During this period clinicians were implementing a new type of treatment, which should be taken into account when interpreting the data and results. It is also important to note that in 2022, when HAT began in Norway, new national OAT regulations came into force, with greater emphasis on user involvement and individually tailored treatment.

The data materials consist of 23 audio-recorded in-depth interviews, which had a mean duration of 82 min. The interviewees were 31 clinicians: 21 nurses (including two social educators with nursing tasks), four medical doctors, three social workers, a psychologist, and two clinic leaders. This multi-disciplinary composition resembles HAT internationally, with the majority of staff being nurses [9, 22, 25]. Nurses were interviewed in groups, and other professionals were interviewed individually, except for two interviews with pairs of social workers and medical doctors.

Interviews were conducted separately with each type of profession to enable in-depth conversations, and to cover the specific roles and tasks of the main professionals involved in HAT. We aimed to identify experiences and views that were widespread across the different groups. The first interviews took place two to four months after the HAT program’s launch, and the second interviews occurred approximately one year later after the staff had adjusted to the new program. Most participants were interviewed twice to capture a broader set of experiences but without any intent of longitudinal analysis. The second round of group interviews involved a few new nurses, while some nurses were unable to take part the second time. Interviews were conducted in the HAT clinics by the first author, a social science researcher without clinical work experience.

The semi-structured interview guide covered various topics, such as views about the medication and questions like: “What is the most demanding part of everyday work in the clinic?” This study was limited to responses about the positive and negative aspects of HAT. No demographic data were gathered, apart from the participants’ profession.

#### Data analysis

We employed an interpretative phenomenological approach [29], oriented towards capturing individual lived experience [30]. After data collection, the first author conducted an inductive thematic analysis [31] of the verbatim transcribed interview data to identify what clinicians experienced and viewed as the main rewards and challenges of providing HAT. This involved a three-step reflexive coding process using the NVivo software: first, reading and coding three selected interviews to develop a codebook; then the codebook was used to code all interviews; finally, these codes were organized and merged into overall themes that captured the most prevalent patterns in the data, as reported below.

## Results

We identified three aspects of treatment provision that were seen as particularly rewarding and three that were experienced as the most challenging. This structure of identified themes is used below to report the results (Table 1).

**Table 1:** Most rewarding and challenging aspects of providing HAT

	Treatment aspect	Involves	Concerns
Rewarding	Observed harm reduction	Observed patient somatic, psychosocial, and behavioral improvements	Treatment outcome
Rewarding	Provision of holistic care	More than medication, everyday meaning, possibility for flexible provision, having a positive impact on patients’ lives	Treatment content and outcome
Rewarding	Positive clinic milieu and patient–clinician relationships	Rewarding therapeutic and informal interactions in a supportive clinical setting	Work conditions and interpersonal relationships
Challenging	Dosing and overdose risk	Frequent enquiries for increased dosage, overdose risk because of concurrent non-prescribed drug use, securing a medically safe treatment	Medical considerations and safety
Challenging	Rule enforcement and aggression management	Negotiating frustration over rule enforcement, employment of sanctions, and handling aggressive behaviors	Program security and interpersonal relationships
Challenging	Treatment beyond medication and harm reduction	Difficult to achieve outcomes beyond harm reduction, uncertainty about treatment value for a small number of the most unstable patients	Treatment content and outcome

### Rewards of providing HAT

Three aspects of providing HAT were identified as the most rewarding to participants: observed harm reduction, provision of holistic care, and the positive clinic milieu and patient–clinician relationships.

#### Observed harm reduction

Participants emphasized the treatment’s importance by describing the harm reduction outcomes they observed. This was important to clinicians, as HAT was new, and these observations were seen as evidence of the treatment’s utility. When asked what HAT is, Daphne answered: “It is clearly harm reduction, and it’s an entry point into treatment.” Regarding whether HAT is mostly harm reduction or treatment, Richard said: “It’s probably like seventy-thirty now. 70% harm reduction.” Participants, like Vicky, gave numerous examples of harm reduction outcomes: “We have observed positive tendencies for those who made efforts to improve their health. Somatic health, primarily. We have seen some change from using intravenously to intramuscular injection, and we hope that maybe more will choose heroin in tablet form”. If patients were to remain in HAT for many years, clinicians saw it as important that they should offer less harmful administration routes.

Donovan, who had no previous experience in harm reduction initiatives, explained how her outlook changed after beginning HAT work:

It’s quite a different way of thinking when it comes to harm reduction and all that […] when we first heard about the [HAT] project, we were very skeptical. Like, ‘Oh my God, are we really going to start this?’ But I think it’s incredible how one’s perspective can change. That you feel we’re really helping the most hard-to-reach patients.

John described further harm reduction outcomes: “They say they can finally wake up and relax without stressing about having to do things to get drugs. For the first time, they could eat breakfast.” For Rebecka, these observations were moving: “I felt this warmth in my chest. It was so beautiful. […] you can clearly see that people are getting an increased quality of life.”

Clinicians also told ‘success stories’ of greater changes for patients, but basic harm reduction outcomes were seen as most widespread and important. Mike emphasized that even if some patients’ lives continue to be highly unstable and they “continue using street drugs”, HAT was still important for reducing harm: “We have received those who haven’t benefited from anything else, who haven’t been able to follow through with any treatment, but they manage to do it here and achieve a bit more stability despite the chaos.” Observed harm reduction outcomes was the most mentioned reason for believing in HAT. It further influenced Richard’s view: “We are very much in agreement that it [HAT] was a much-needed service and that it should continue.”

Consistent with previous findings [16, 7], the positive harm reduction outcomes they saw in patients validated and gave meaning to the participants’ work; the positive changes were described by some as both “reassuring” and “inspiring.” Similarly, clinicians in traditional OAT have expressed increased motivation to provide treatment after observing positive patient outcomes [32]. However, these harm reduction outcomes may seem to stand in contrast to the potential risk of harm from overdoses that are described as a challenge of HAT below.

#### Provision of holistic care

Participants described treatment provision as rewarding due to its holistic nature, which went beyond medication and addressed individual patient needs. Some clinicians viewed HAT as an anchor point for patients, providing a comprehensive framework of care that combined various forms of support within the program and facilitated connections with psychosocial services outside the program.

While many of the reported harm reduction outcomes resulted from provision of medical heroin, participants repeatedly underlined the importance of providing holistic treatment. Matilda was surprised by experiences that went beyond harm reduction: “I find it surprising that we already have a patient at Hekta på jobb [an individual job placement and support initiative], and that some patients are getting better as quickly as they are. Suddenly, they start talking about things that matter in life, in just a few weeks.” Although patients who started job training were considered outliers by clinicians, these examples seemingly demonstrated the treatment’s broader potential for contributing to patients’ social integration.

Patient involvement in their own treatment decisions was also mentioned as a difference from traditional OAT. The level of user involvement in HAT contributed to the clinicians’ sense of delivering a holistic patient-centered treatment. Eira explained that: “I feel that the job is very valuable. For the patients, you know. Allowing them to be so actively involved in decisions, as they are here, feels extremely rewarding.”

Vicky, who had significant clinical experience with traditional OAT, said it was rewarding to hear patients’ positive feelings about HAT: “It’s the overall satisfaction that the patients convey. Many have said: ‘Now… I have a new life. Finally, I can relax. Finally, I can feel normal.’ We hear that constantly. We rarely hear this in traditional OAT.” Several participants described HAT as better than traditional OAT. This type of patient feedback apparently demonstrated HAT’s wider impact and difference from traditional treatment options, which gave the clinicians reason to believe in HAT.

For many participants, like John, belief in HAT was reinforced by the fact that they provided services and support beyond medication, addressing: “other needs that the patients have, such as social needs, NAV [a public social service], and dental care.” Charlie described the benefits of the support services they offered: “There are changes going on for many, I think. And, of course, things don’t happen overnight. But surprisingly, many are undergoing a change in terms of both health, social situation, and hygiene.” He further added: “It seems like they have gotten more future-oriented plans, like, ‘This is something I might be able to achieve someday.’ Instead of going downtown and engaging in all the activities they used to do there.”

Participants, like Nicola, gave various examples of encouraging patient changes related to the psychosocial support they offered: “They are taking the initiative and asking, ‘Can you call NAV and the general practitioner, and can you arrange this or that.’ They simply want predictability. So, things are happening.”

Mike emphasized the importance of psychosocial assistance as regards believing in HAT: “We may have managed to get a little closer to patients and help them a little more on their way than if they were at other places that perhaps lack the support we have.” Clinicians also helped patients to remember and follow up on various appointments with health, social, and housing services. Richard described the HAT role for patients as an “anchor point” in many patients’ lives. The psychosocial support and care that clinicians provide include coordination with external services.

Providing holistic care was seen as rewarding because it allowed clinicians to better meet patients’ individual needs beyond medication and provided them with room for agency in treatment provision. This aligns with findings from other studies [16]. These qualities of HAT seemingly contributed to clinicians’ support for the treatment. This is important, as treatment outcomes are likely to improve when clinicians believe in the treatment they provide [1]. Seeing positive outcomes of holistic care has been reported to be a particularly gratifying aspect of HAT work [32]. However, contrasting experiences emerge in the challenges described below, particularly in achieving care that extends beyond medication and basic harm reduction.

#### Positive clinic milieu and patient–clinician relationships

The participants emphasized that positive relationships with patients and good clinic atmospheres were important to their work experiences. They described patient–clinician interactions as positive and a crucial component of their everyday treatment provision. Many expressed being surprised that HAT was operating better, and with less patient conflict, than they had initially expected.

Positive patient interactions were clearly important, as Denise explained: “Overall, I have been very satisfied. It’s nice to work with satisfied clients. From the beginning, there was quite a positive atmosphere”. In a group interview, clinicians were eager to describe their relationships with patients:

Nicola: Overall, I find it very pleasant with the patients.

John: We have developed quite close relationships with many. Many of them sit down, open up, and want to talk.

Donovan: We laugh and joke a lot, too.

After injecting their medication, patients are required to remain in the clinic’s observation room for at least 20 min, where they have access to newspapers, magazines, and hot drinks. The Oslo clinic also served simple meals. This space was described as important for building rapport with patients. Ruby described these elements as important to the clinic milieu: “In terms of the atmosphere here, it makes sense to have some small rituals. They can have a coffee and read the newspaper, which makes it sensible to spend half an hour here.” Olivia said: “I find the atmosphere in the observation room quite surprising”, then explained that the patient–clinician interactions which took place there: “could almost become a form of milieu therapy.”

Patients’ twice-daily visits to the clinic involved a unique opportunity to build patient–clinician relationships through frequent interaction, in contrast with traditional OAT, according to Denise:

Compared to [traditional OAT], I feel that they get to know us differently here. There, patients primarily interact with one clinician, right? Whereas here, we are all actively involved, and they encounter every staff member twice a day, so I feel that everyone forms relationships with everyone else, albeit in a different way.

When asked whether HAT differs from other addiction treatments, Vicky responded: “HAT is different. I find that we have much closer contact with the patients. We talk to almost all of them, every single day. We can follow up much more closely.” Matilda further explained the advantages and importance of this close relationship: “I see the positive aspect of us being able to be so closely involved. We can notice any changes or if there is something we are worried about, whether it’s somatic or if they are feeling unwell. I believe that can be somewhat crucial for this group.” These close relationships were described as crucial to the clinicians’ ability to provide the holistic, patient-centered care described above.

Many participants had expected to face more interpersonal conflicts with patients before starting HAT work. Several compared HAT with traditional OAT: Vicky, for example, said: “I haven’t experienced as much… how should I put it? The trouble, anger and agitated patients that are often observed in traditional OAT clinics”. Clover commented regarding her work with HAT patients, “I think the collaboration with the patients is beyond my expectations.”

Participants saw the positive clinic milieu and patient–clinician relationships as rewarding. This was strongly related to their working conditions, and not primarily about treatment content and outcomes, as described in the sections above. Previous studies have also found that HAT staff emphasize their relationships with patients as making their work valuable, and a reason why they remain in this type of work [16]. The opportunity to build therapeutic alliances with patients may further inspire positive attitudes toward HAT among clinicians [26]. But along with these positive interactions there were also difficult and unpleasant ones, as described in the section below on rule enforcement and aggression management.

The three rewarding aspects of providing HAT described here all seemingly added positive meaning to participants’ clinical work and created enthusiasm about HAT.

### Challenges of HAT provision

Three aspects of providing HAT were identified as being particularly challenging: dosing and overdose risk, rule enforcement and aggression management, and treatment beyond medication and harm reduction.

#### Dosing and overdose risk

A core challenge was safely providing medical heroin, particularly because concurrent use of non-prescribed drugs heightened patients’ overdose risks. Dosing and dose adjustments were also complicated.

Participants found patients’ frequent requests for increased dosages of medical heroin to involve a difficult balancing act between accommodating patient preferences and ensuring medically safe treatment. Vicky said: “They know they can always discuss their dose, and we hear that constantly. The thing is that most discussions are about the dose… and the patients may want a slightly higher level of effect than we allow”. Clinicians use a standardized observational scoring tool to assess medical effects, Vicky continued, but: “the patient’s subjective experience of the effect is a bit different. And that’s where some conflicts or disagreements arise”.

Darcy explained how the dosage a patient had been receiving for some time could also suddenly become too much: “We expected that a dose that has been tolerated just fine for several weeks suddenly may not be tolerated as well. Then we assume it’s due to the use of other substances simultaneously”. Regarding dosing challenges, George described wide variation in how patients were affected: “Some are abstinent when they arrive, they enter to get their injection, and they nod off for a few minutes, and then they are back. Others are really on their knees, and I am really worried: ‘Is the dose right? Should it have been lower?’”.

Differences in medical effects were partially related to dosage differences, which were adapted to patients’ individual situations and needs. However, their non-prescribed drug use was equally concerning, said George: “It has to do with what they have taken before coming here. I sometimes wonder if this is being investigated thoroughly enough before they get the injection.” Isla gave a related example: “Sometimes you don’t see it [non-prescribed drug use] until they suddenly overdose. We’ve had one or two cases in the last few weeks.” Patients’ use of non-prescribed drugs was contingent, sometimes increasing the challenge, according to Mike: “Especially when they have received their paycheck, they may engage in more substance use than usual”.

Many participants, like Vicky, said: “We have different groups of patients. Some are not using substances other than heroin, and they are completely fine and stable. They use the same dose over time, are not seeking to be heavily influenced, and just want to get well.” However, another group of patients presented more challenges. She continued, “We also have patients who use additional drugs. […] They may have a period when they use more amphetamines. During these times, we see that they cannot tolerate their usual heroin dose. So we have to reduce it.”

Non-prescribed drug use was seen as the major challenge to safe dosing and preventing overdoses. These challenges were particularly acute with the most unstable patients. A specific dilemma was highlighted for this group by Daphne:

What we’re experiencing and hearing a lot is that they are dissatisfied because their doses are too slowly raised after we reduced them. They feel they don’t get enough [in HAT], which leads them to buy street heroin, and at the same time, they are too influenced by the street heroin for us to increase their dose because they score too heavily after an injection. It’s a delicate balance.

Because medical heroin is the main component of HAT, it is unsurprising that participants had significant problems ensuring medically safe, secure treatment for patients who also use street drugs. The challenge of this “delicate balance” and related concerns have also been reported in previous studies of HAT clinicians [22, 23]. A key part of the dilemma for clinicians is balancing and negotiating considerations of patients’ needs versus a medically safe treatment program [7]. The diverse levels of clinical challenges directly related to patients’ degree of stability/instability are not, to our knowledge, identified in previous studies on clinical experiences with HAT.

#### Rule enforcement and aggression management

Enforcement of rules governing the treatment program and patients’ behavior in the clinics was reported as a recurrent source of frustration and aggressive outbursts among patients. This also included the employment of sanctions when rules were violated. Rule enforcement and aggression management were seen as core challenges of providing HAT.

Darcy gave an example: “We just had a case, only about an hour ago, where the patient arrived heavily intoxicated and with what we considered an uncomfortable appearance, leading us to have a discussion and then ultimately deciding not to administer the dose.” A breach of rules led to the denial of medical heroin. The clinicians then took steps to prepare for an aggressive response, he continued: “We chose to call security as a precaution because we thought there could be aggressive behavior. It turned out fine, but at that moment, you feel a bit like, ‘Oh, this could…’. It’s a bit tense.” Even if this kind of serious incident was described as relatively rare, it featured as a key challenge for clinicians.

To the question about how they had handled acting out incidents, Richard replied: “It has turned out fine so far. But of course, it’s fragile. And I am always waiting for something to happen.” Rebecka described an unpleasant incident: “Last week, I felt a bit… not scared, but it felt a bit unsafe because it was so turbulent here. Many were intoxicated and angry.” The level of intoxication and instability could correspond with the level of challenge with certain patients. She added further negative experiences: “I thought: ‘Now I’ve reached my limit’. I maybe should have slammed the door shut and locked it, because that wasn’t acceptable. Having someone in your face angry and almost spitting at you.” Several clinicians described discussions of such incidents, as well as other security issues and aggression management, in staff meetings and during training. A known history of violent behavior was also an exclusion criterion when clinicians considered who was eligible for HAT.

While different forms of patient aggression and frustration were described, there were no experiences of serious interpersonal violence. Anger could also be triggered by events outside of HAT, said Mike: “I do experience a bit of acting out, but usually, it’s not directed toward me, as there’s usually someone out there involved”. Outbursts were often directed at social services or could be a response to negative events, like having been robbed.

Participants mentioned disagreement among clinicians about what amounts to risk and aggression. Some described open doors inside the clinic as a sign of trust between patients and clinicians, while others like Clover emphasized the risk it posed: “If something happens, we have ourselves to blame because we have all the doors open”.

Clinicians described discontent among patients due to perceived differences in how staff behaved toward them. Patients could feel they were treated unfairly or discriminated against. Responding to this frustration was challenging, as clinicians had differing views on what constituted acceptable and tolerable patient behavior. For example, Ruby described how clinicians might enforce rules differently: “Regarding the injection room, it is likely that different approaches have been taken. Some [clinicians] may turn their backs and let them [patients] do their thing, while others may monitor and actively keep an eye on things”. Rebecka spoke of how different interpretations of rules could reinforce patients’ feelings that they are being treated differently: “It can potentially be a problem if patients feel that we are very different, that ‘She is nice, but he is not.’ We need to have a common approach to avoid these experiences for patients”. This issue may have become less pressing as clinical routines have become more streamlined, but with close interactions and individualized treatment, the challenge is likely to endure.

Participants described varied challenges with rule enforcement and management of patient aggression. Differences in clinicians’ rule and sanction enforcement, and how they understood and reacted to incidents of aggression, added to these challenges. Similar challenges have appeared among HAT staff in other studies [25]. The enforcement of rules and sanctions can sometimes also negatively impact the therapeutic alliance between clinicians and patients [32]. This contrasts with the positive clinic milieu and relationships between clinicians and patients, described above as rewarding.

#### Treatment beyond medication and basic harm reduction

Participants faced significant challenges with achieving treatment outcomes extending beyond basic harm reduction. While they emphasized the importance of holistic care, they experienced barriers that limited their ability to offer more than medication.

These challenges were influenced by the varying degrees of patient engagement with psychosocial assistance. According to Mike: “One can already see that some are able to engage more in activities, while others are here primarily for the sake of their medication.” Becoming too affected by medication could even conflict with clinicians’ opportunities to establish a dialogue about further psychosocial support. Mike said: “The obstacle can be that, at certain times, they can be very intoxicated, and then it’s difficult to establish a good connection with them.” As with the two challenges above, participants referred to different categories of patients, from the most stable to the most unstable, giving examples of how the degree of challenge increased according to the level of instability.

Richard described HAT outcomes for patients: “We haven’t come too far in thinking about, like, perhaps rehabilitation for those who want it. Some are still at a point where they want to use the substance they need to feel well, but HAT could perhaps be a step toward further rehabilitation.” While participants observed positive treatment outcomes, Richard and others said: “I observed quite a quick, positive change in their lifestyle, how they presented themselves, and their overall wellbeing. […] However, I’ve noticed that some of them seem to have come to a standstill after that.”

On the issue of staffing and the opportunity for additional treatment beyond medication, Daphne insisted: “I think that if we had more staffing, we could probably do more. […] but it takes time, and we shouldn’t expect our patients to immediately, once they are stabilized on a heroin dose, to think, ‘Now I’m ready to do something more’.”

Limited staffing could restrict what clinicians were able to provide and the time they had available for patient follow-up. Nurses described how limited staffing restricted their efforts beyond the medication, which was prioritized. Ruby said: “Everyday nursing becomes routine with the administration of medications, and it takes a lot of capacity”. Olivia elaborated the challenge: “Because we see them so often, we get to know them well. We want to be available for them, and this is the only treatment option they have… but it is very limited what we can actually do for them.”

Reflecting on how they assisted patients in finding new organized activities, Daphne said: “It’s going a bit slow; it’s difficult to motivate patients for it. So, it’s a challenge, but we know that it takes time to change a life. […] I do see that something has changed. But we don’t have a lot of patients in regular activity.” Barriers to assisting patients beyond medication were sometimes caused by actors external to HAT, like public services, housing, or third-sector initiatives. Daphne described what they missed: “It’s not HAT alone that can change a life. Other agencies need to get involved as well, and… I believe that if someone is to have a better life, we must think a bit bigger and think more in collaboration with others.”

Initiating treatment beyond medication and achieving outcomes beyond basic harm reduction was seen as challenging. Relatedly, clinicians spoke of ongoing insecurities and discussions about the aims of HAT, and whether it should be primarily harm reduction or something more. Setting aims would also become a criterion for deciding whether what they achieved with patients was in line with their aims. Discussions about the specification and operation of HAT goals and whether those goals should primarily be harm reduction or encompass broader objectices have also been pressing issues for HAT staff in other countries [23].

## Discussion

The findings outline aspects of HAT provision that participants found personally rewarding and important for patients. At the same time, their work involved continuous negotiation of challenges. Providing HAT involved a combination of positive and challenging experiences, and the tensions between them. The challenges clinicians faced when providing HAT varied according to the patient group involved: patients differed in the instability of their everyday lives. Participants said that while most patients rarely caused major workday challenges, a minority took up significant time and energy. They struggled most with a small subgroup of the most unstable patients, for whom they were also uncertain about the treatment’s utility. The most unstable patients were generally viewed as presenting the most difficulties across all the challenging aspects of HAT provision.

The different rewards and challenges we found may seem contradictory, and they involve tensions between overlapping positive and negative experiences. First, the rewards of providing holistic care seemingly conflict with the described challenges of achieving outcomes that go beyond medication and basic harm reduction. As regards the rewards of providing holistic care, the early trial status of Norwegian HAT and the unconsolidated aims of HAT among participants, may have contributed to them setting over-ambitious goals for HAT and on behalf of patients. The treatment outcomes they observed, however, seemingly fell short of these. This tension may perhaps reflect a discrepancy between participants’ early treatment ambitions and the actual outcomes. This highlights the need for setting clearer and prioritized aims for HAT, configuring treatment to align with those aims, and assessing results in the light of them.

A second tension existed between descriptions of a good atmosphere in the clinic and positive relationships with patients, as against the challenges of managing aggression. While these two sides of HAT are contradictory, such a situation is common in relationships and social life generally. Aggression issues were mainly related to the enforcement of clinic rules and disagreements over dosing, while other types of interaction were described as positive. It is, therefore, unsurprising that clinicians experienced both rewarding and challenging interactions with patients. However, it does underline the importance of efforts to reduce conflicts related to rule enforcement and dosing.

Third, there is an apparent tension between the harm reduction outcomes valued by participants and the potential risk of harm from overdoses when providing medical heroin to poly-substance-using patients. This illustrates the delicate balance of benefits and risks that clinicians constantly have to navigate, similar also to other types of OAT [33].

These dualities found in clinicians’ experiences are also reflective of an overarching tension in HAT globally: while holistic and patient-centered care calls for individual adjustments and flexibility to tailor treatment to each patient’s needs and preferences, the risk of overdosing and diversion of medical heroin necessitates precautions and consistent rule enforcement to ensure program security and patient safety [see also 26]. This duality, of consideration for patient-centered care on one hand, and of program-centered care on the other, is inherent to HAT [28].

The participants, while implementing the new treatment program, emphasized the importance of establishing clinic rules and making them clear to patients to facilitate enforcement. At the same time, these clinicians valued flexibility and individual consideration to tailor treatment to patients’ needs. Efforts to reduce rigid enforcement and enhance flexibility for patients also figure in other addiction treatments, and such ‘negotiated flexibility’ seeking to provide patient-centered care is a feature of other HAT programs [7, 34].

The overarching tension in HAT, between providing patient-centered care and upholding program security and safety, involves difficult considerations and decision-making [see also 35]. It is, therefore, a core issue that needs to be acknowledged and attended to by HAT clinicians and leaders both in Norway and worldwide. Measures should be developed to support clinicians’ negotiation of these tensions. This may be particularly important for newly established HAT programs or trial projects, like the Norwegian one, where clinical procedures, rules and aims are potentially more contingent and negotiable.

Unlike permanent programs, Norwegian HAT is still a trial project. The trial period lasts until the end of 2026, and a government decision about its future is expected earlier in 2026. The uncertainty created by the program’s temporary status may have negatively influenced clinicians by creating an atmosphere of uncertainty [36]. Differences in HAT’s status, configuration and funding across countries determine what treatment clinicians can provide. Such key factors impact treatment scope, content, and quality [35, 37]. For example, limited state funding at times restricted the number of patients that could be enrolled in Norwegian HAT clinics. Even so, several participants still described HAT as “better” than traditional OAT, seemingly partly because HAT was better resourced, with more staff per patient and better opportunities for follow-up on site by daily encounters with patients [see also 11].

Differing national configuration, funding, and legal regulation of HAT programs seem particularly important to examine in future studies to help identify and explain the factors that lead to differences in the content, quality, and outcomes of HAT across countries, a topic which has been given little scholarly attention [but see 38].

Prior experience of providing addiction treatment varied widely among the Norwegian HAT staff. Structured training and opportunities for specialization appear important for ensuring the quality of HAT [25]. The patient–clinician relationship in addiction treatment is crucial to patient satisfaction with treatment and its outcomes [17, 39]. Clinical task and skills training does not guarantee that clinicians can engage with patients therapeutically or in ways that help establish trusting relationships [40]. The fact that HAT staff hold the role as ‘gatekeepers’ to medication and services in HAT may create barriers to therapeutic alliances, for example if patients perceive restrictions as unfair sanctions [32, 41]. Training clinicians to manage this role could include ongoing self-evaluations of their practices regarding restricting patient access to the services provided, and awareness-raising about how they manage their power as gatekeepers [see also 21, 42]. It also appears important to train clinicians to apply more streamlined enforcement of clinic rules and sanctions, in order to avoid unequal patient treatment on issues unrelated to medical considerations or individual treatment adjustments.

This study was not without limitations. Data were unavailable to distinguish between experiences among different professional groups, or their backgrounds and experiences related to addiction and addiction treatment before HAT work. Emphasizing these factors might provide insights into the relations between professional background and/or work experience and different HAT-related experiences. It is further important to note that Norway’s HAT was in its early phase throughout the study, likely impacting the data and results.

Despite these limitations, the results shown here are the first published on clinicians’ roles in, and experiences of, providing HAT in Norway. The participants in this study were further representative of the professions typically working in HAT internationally, with the majority being nurses [9]. Results provide important knowledge relevant to the international administration of HAT generally.

## Conclusions

The study makes clear how and why clinicians believe in HAT’s feasibility and utility, as well as the challenges of running the program itself and achieving outcomes beyond harm reduction and basic medication for patients. While studies about clinical experiences of HAT have usually examined individual or limited aspects of treatment provision, this study provided an overview of the main aspects of the rewards and challenges of providing HAT. Importantly, it also showed the tensions between these overlapping and sometimes contradictory aspects of HAT provision. This included the “delicate balance” involved in frequently having to weigh considerations of patient needs and preferences against their safety and program security – a dilemma inherent to HAT. Our results both confirm and expand on the findings of previous studies, suggesting that the rewards and challenges identified here, together with some of the tensions between them, are likely transferrable to HAT programs in other countries. The outlined clinical rewards and challenges of HAT could serve as a foundation for comparative studies investigating the same dimensions of HAT provision across countries. Based on the findings, we have also suggested measures for current and future HAT programs to support clinicians in navigating the potentially contradictory considerations inherent in their daily work and decision-making.

## Data Availability

The interview transcripts generated during the interviews in this study are not publicly available to preserve the confidentiality of the participants.

## Abbreviations

HAT: Heroin-assisted treatment
OAT: Opioid agonist treatment
OUD: Opioid use disorder
